# Domestic Correspondence

**Published:** 1891-02

**Authors:** 


### Domestic Correspondence.

TO THE EDITOR:

Will you kindly insert the following sets of resolutions passed, respectively, by the Massachusetts Dental Society and the New England Dental Society at the Union Meeting held last October in Boston.

Very truly,

EDGAR O. KINSMAN,
*Secretary M.D.S. and N.E.D.S.*

Resolutions by the Massachusetts Dental Society upon the death of Gustavus A. Gerry, D.D.S.

WHEREAS, Gustavus A. Gerry, D.D.S., of Lowell, Massachusetts, has been called by death from his earthly labors to his future reward. To the various associations with which he was connected in his life-work, and especially to the Massachusetts Dental Society, in which he held for many years an active and honorable membership, discharging an important trust in the capacity of president, his loss will be most seriously felt. Therefore be it

*Resolved*, That we deplore his early demise and the loss we as a society have sustained. His eminently good, sound qualities, his integrity of character in his professional relations, made him a desirable friend in all the walks of life.

*Resolved*, That our sympathy be extended to his family in their loss of a true husband and a kind father.

Signed, W. E. PAGE,
JOS. KING KNIGHT,
S. G. STEVENS,
*Committee.*

Resolutions passed by the New England Dental Society upon the death of Gustavus A. Gerry, D.D.S., of Lowell, Mass.

WHEREAS, Once more we are called to mourn the loss of a beloved brother who, for so many years, has been our companion, co-worker, and friend. His pleasant smile, his words of encouragement, many graces of character, and a life of usefulness and honor make his memory a delight to us.

WHEREAS, His record is marked with fidelity, uprightness, and sincerity, therefore be it

*Resolved*, That in the death of Dr. Gustavus A. Gerry, of Lowell, Massachusetts, this society loses an active member, whose faithful earnest work is known throughout the profession.

*Resolved*, That this expression of our regret for his loss be entered upon the records of this society, and that a copy of these resolutions be sent to his afflicted family.

Signed, C. A. BRACKETT,
A. H. GILSON,
R. R. ANDREWS,
*Committee.*

---

TO THE EDITOR:

The Twentieth Annual Meeting of the Kansas State Dental Association will be held at Wichita, Kansas, Tuesday, April 28, 1891, continuing four days.

The profession is cordially invited to meet with us.

C. E. ESTERLY,
*Secretary.*

LAWRENCE, KANSAS, January 8, 1891.

---

### NEW ENGLAND DENTAL SOCIETY.

TO THE EDITOR:

Will you kindly insert the following list of officers of the above society in the INTERNATIONAL DENTAL JOURNAL, elected at the last annual meeting, held in Boston, October, 1890: President, Dr. W. E. Page, Boston, Mass.; First Vice-President, Dr. J. F. Adams,

Worcester, Mass.; Second Vice-President, Dr. S. G. Stevens, Boston, Mass.; Secretary, Dr. Edgar O. Kinsman, Cambridge, Mass.; Assistant Secretary, Dr. J. H. McQuade, Medford, Mass.; Treasurer, Dr. G. A. Young, Concord, N. H.; Librarian, Dr. A. H. Gilson, Boston, Mass.

*Executive Committee.*—Dr. C. W. Clement, Manchester, N. H.; Dr. George F. Cheney, St. Johnsbury, Vt.; Dr. J. E. Quinn and Dr. J. H. Daly, Boston, Mass.

EDGAR O. KINSMAN,
*Secretary.*
15 BRATTLE SQUARE, CAMBRIDGE, MASSACHUSETTS.

---

TO THE EDITOR:

*American Academy of Dental Science.*—The Twenty-third Annual Meeting of the American Academy of Dental Science was held at Young's Hotel, in Boston, on Wednesday, November 12, 1890.

The chair was occupied by the president, Dr. F. N. Seabury, of Providence, R. I.

The corresponding secretary and the treasurer presented their annual reports, which showed the society to be in a very prosperous condition.

Seven new members have been admitted during the past year, —viz., W. H. H. Thackston, M.D., D.D.S., of Farmville, Va.; William S. Sherman, M.D., D.D.S., of Newport, R. I.; George T. Baker, D.D.S., of Boston; H. S. Draper, D.D.S., of Boston; George H. Payne, D.D.S., of Boston; William Y. Allen, D.D.S., of Boston; and Geo. C. Ainsworth, D.D.S., of Boston.

The following officers were elected for the ensuing year: President, Dr. F. N. Seabury, of Providence; Vice-President, Dr. C. A. Brackett, of Newport, R. I.; Corresponding Secretary, Dr. Edward N. Harris, of Boston; Recording Secretary, Dr. Charles H. Taft, of Cambridge; Treasurer, Dr. V. C. Pond, of Boston; Librarian, Dr. Edward C. Briggs, of Boston.

*Executive Committee.*—Drs. Eugene H. Smith, Thomas Fillebrown, and F. E. Banfield, of Boston.

Members deceased during the past year: Drs. D. S. Dickerman, of Taunton; W. A. Bronson, of New York; Frank P. Abbott, of Berlin; E. B. Gardette, of Philadelphia; Henry J. Bigelow, of Boston; and G. A. Gerry, of Lowell, Mass.

Resolutions of respect to their memory were adopted.

The annual address was delivered at 4 o'clock P.M., by W. H.

H. Thackston, M.D., D.D.S., of Farmville, Va., ex-president of the Southern Dental Association, and he chose for the subject of his oration, "American Dentistry."

He was followed by Charles H. Taft, A.B., D.M.D., of Cambridge, with an address upon "The Progress of Science and its Influence on Modern Civilization." Both addresses were listened to with deep interest, and at their close elicited prolonged and hearty applause from all present.

Votes of thanks were given to Drs. Thackston and Taft, and copies of each address were requested for publication in pamphlet form. The Academy then adjourned at 6 o'clock to partake of the anniversary dinner. The party broke up at ten o'clock with many expressions of pleasure.

EDWARD N. HARRIS, D.D.S.,
*Corresponding Secretary.*

2 PARK SQUARE, BOSTON.

---

TO THE EDITOR:

I have before me a circular from Claudius Ash & Sons, of date November 20, stating that *they will be unable to import any further shipments of their teeth*, that they shall have to decline executing any further wholesale orders, and that the profession in this country can only be supplied until their present stock is exhausted.

The necessity for the above lies in the provision of the McKinley Tariff Bill, raising the duty on plain pin teeth from $17.50 to $52.50 per thousand.

It is a matter of serious interest to the profession to know the details of the McKinley Bill, so far as they effect dental materials. As a matter of news can you not give us this in an early issue of your magazine?

English teeth have special qualities all their own, appreciated by makers of metal plates, and by bridge- and crown-workers, and if it is the policy of our government to absolutely prohibit the entry of foreign materials necessary for our use as a profession, we ought to know it.

It would be a very interesting piece of information to know the names of the beneficiaries to whom the profession is indebted for the exclusion of English teeth.

Yours very truly,

CHAS. F. ALLAN.

NEWBURGH, N. Y., December 8, 1890.

To the Editor:

Sir,—It is a noticeable, as well as a somewhat painful, fact that certain individuals who attend the meetings of societies are in the habit of consuming much of the time allotted to gentlemen present for speaking by lengthy harangues, or speeches long drawn out, when the substance of all they impart could be easily summed up in a comparatively few words. As a result, those who are compelled to listen become impatient or weary, and some who are desirous of making remarks are debarred, and find no opportunity for speaking before the hour of adjournment is reached. Why cannot gentlemen be more considerate? If they have anything to say, say it in as clear and concise a manner as possible and give other's a chance to do likewise. Do not take hearers around Robin Hood's barn forty times before showing them how you enter, even though you have only a frog to exhibit. Get at the pith of your subject at once, without giving utterance to preliminary showers of irrelevant verbiage. Remember, dear time-monopolists, that "brevity is the soul of wit."

